# Sub-threshold signal encoding in coupled FitzHugh-Nagumo neurons

**DOI:** 10.1038/s41598-018-26618-8

**Published:** 2018-05-29

**Authors:** Maria Masoliver, Cristina Masoller

**Affiliations:** Departament de Fisica, Universitat Politecnica de Catalunya, Rambla de Sant Nebridi 22, 08222 Terrassa, Barcelona Spain

## Abstract

Despite intensive research, the mechanisms underlying the neural code remain poorly understood. Recent work has focused on the response of a single neuron to a weak, sub-threshold periodic signal. By simulating the stochastic FitzHugh-Nagumo (FHN) model and then using a symbolic method to analyze the firing activity, preferred and infrequent spike patterns (defined by the relative timing of the spikes) were detected, whose probabilities encode information about the signal. As not individual neurons but neuronal populations are responsible for sensory coding and information transfer, a relevant question is how a second neuron, which does not perceive the signal, affects the detection and the encoding of the signal, done by the first neuron. Through simulations of two stochastic FHN neurons we show that the encoding of a sub-threshold signal in symbolic spike patterns is a plausible mechanism. The neuron that perceives the signal fires a spike train that, despite having an almost random temporal structure, has preferred and infrequent patterns which carry information about the signal. Our findings could be relevant for sensory systems composed by two noisy neurons, when only one detects a weak external input.

## Introduction

In spite of having been the object of intensive research for decades, the mechanisms used by neuronal populations to efficiently encode and transmit information, in noisy environments, remain poorly understood. Advances in this area are crucial, not only for understanding brain function, but also, for developing artificial intelligence systems^1^ and even photonic neurons that could revolutionize the field of optical information processing^2–4^.

Various mechanisms have been proposed to explain how neurons encode external inputs, which can been viewed as complementary, or functional, under different situations^5–10^. Neuronal populations can encode information in the spike rate, in the spike timing, in the frequency content of spike sequences, in the spatial coherence of the spikes, etc., and measures based on information-theory have been used to quantify the information content of spike sequences^11–14^. A lot of research has focused on the statistics of the time intervals between consecutive spikes (inter-spike intervals, ISIs) and how serial ISI correlations affect information encoding^15–21^.

Recently, the response of an individual neuron to a weak periodic signal was studied numerically^22^, in the framework of the stochastic FitzHugh-Nagumo (FHN) model^23,24^. The analysis focused in a weak, sub-threshold signal, which means that the signal alone does not produce spikes. Therefore, without background noise, the neuron’s membrane voltage displays only small, sub-threshold oscillations. However, in the presence of noise, the firing activity of the neuron encodes information about the amplitude and the period of the signal^22^. By analyzing the ISI sequence using a nonlinear symbolic method known as *ordinal analysis*^25–28^, it was shown that the weak periodic signal induces the emergence of temporal ordering in the timing of the spikes, which is absent if the neuron’s firing activity is only due to uncorrelated noise^22,29^. Despite the spiking activity being almost random, temporal ordering was detected in the form of over expressed and less expressed symbolic patterns (referred to as ordinal patterns, defined by the relative timing of the spikes), which depend on the period of the signal and on the level of noise. The pattern’s probabilities depend also on the amplitude of the signal, and thus encode information about both signal features, the amplitude and the period. A resonance-like behavior was found, as certain periods and noise levels enhance temporal ordering, maximizing (or minimizing) the probability of the over (less) expressed patterns.

An open question is whether this encoding mechanism is robust when a neuron is not in isolation. In particular, can a neuron still use this mechanism to encode a sub-threshold periodic signal, when it is coupled to another neuron that does not perceive the signal? To address this question we simulate two stochastic FHN neurons that are mutually coupled, with a periodic sub-threshold signal applied to one of them. Despite lacking a realistic biophysical simulation of neuronal coupling, model simulations yield theoretical insights that suggest that the encoding mechanism is plausible, as the neuron that perceives the weak signal still encodes the signal information in a spike train that has over expressed and less expressed patterns, whose probabilities depend on the signal’s amplitude and period.

## Results

**Figure 1:**
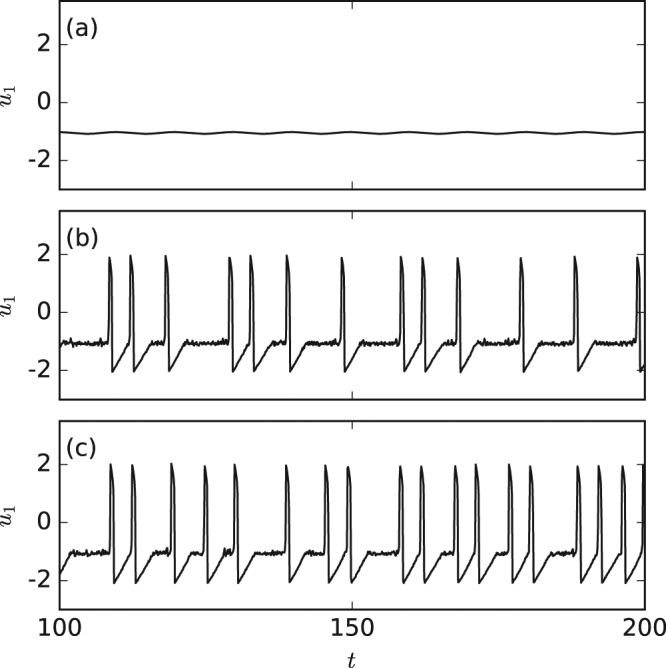
Time series of the voltage-like variable of neuron 1 when (**a**) the signal is applied, and there is no noise and no coupling; (**b**) the signal is applied and there is noise but no coupling and (**c**) the signal is applied and there is noise and coupling. The parameters are *a*_0_ = 0.05, *T* = 10 and (**a**) *D* = 0, *σ*_2_ = 0; (**b**) *D* = 2 ⋅ 10^−6^, *σ* = 0; (**c**) *D* = 2 ⋅ 10^−6^, *σ* = 0.05.

We simulate the coupled FHN neurons as described in *Methods*, with a periodic sub-threshold signal that is applied to one of the neurons, referred to as neuron 1. Figure 1 displays the voltage-like variable of neuron 1, *u*_1_, in different situations. When there is no noise, no signal and no coupling, the neuron is in the rest state and when the sub-threshold signal is applied, *u*_1_ displays small sub-threshold oscillations [panel (a)]; when noise is added, noise-induced spikes are observed, which carry information about the applied sub-threshold signal [panel (b)]; and when the coupling to neuron 2 is added, a noticeable effect is the increase of the firing rate [panel (c)]. The differences that are qualitatively observed in these time series are going to be quantitatively addressed by using the methods of analysis presented in *Methods*.

**Figure 2:**
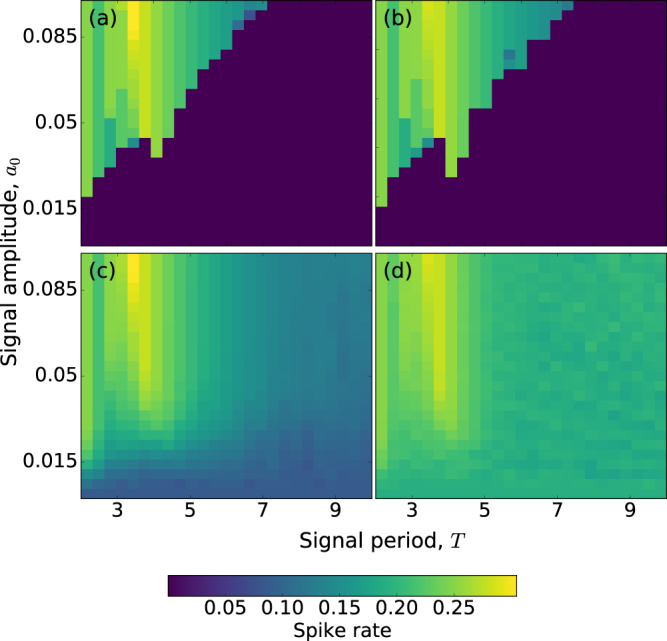
Influence of the signal parameters in the spike rate. The spike rate of neuron 1 in color code is plotted as a function of the signal amplitude, *a*_0_, and period, *T*. Panels (a and b) display the deterministic spike rate (*D* = 0) without coupling (*σ*_1_ = *σ*_2_ = 0) and with coupling (*σ*_1_ = *σ*_2_ = 0.05), respectively. In panels (c and d) the noise is included (*D* = 2 ⋅ 10^−6^).

As we are interested in the encoding of weak signals, we first have to distinguish between a sub-threshold and a super-threshold signal. The first one refers to a signal which, in the absence of noise, it does not induce any spike [*u*_1_ displays small oscillations, as in Fig. 1(a)], while the second one is a signal that is strong enough to induce spikes. A periodic signal can be either sub-threshold or super-threshold depending on both, the period and the amplitude. Thus, to identify the parameters where the signal is sub-threshold, in Fig. 2 we plot in color code the spike rate (i.e., the inverse of the mean ISI, 1/〈*I*〉), as a function of *a*_0_ and *T*. In panel (a) neuron 1 is isolated (*σ*_2_ = 0), while in panel (b) it is coupled to neuron 2 (*σ*_1_ = *σ*_2_ = 0.05).

When the neuron is uncoupled, for large amplitude and/or small period the signal is super-threshold, otherwise is sub-threshold. When the neuron is coupled to neuron 2, we note that the super-threshold region is slightly larger in the parameter space (*a*_0_, *T*), as compared to the uncoupled case.

When we include noise, Fig. 2(c) and (d), we first note that in the super-threshold region (yellow) the spike rate does not change significantly (it is about the same as for *D* = 0). This is due to the fact that in this region the spikes are mainly induced by the signal.

In contrast, in the sub-threshold region, comparing the uncoupled (panel c) and the coupled (panel d) situations, we note that coupling significantly increases the spike rate (it almost doubles). Therefore, in this region coupling plays the role of an extra source of noise.

**Figure 3:**
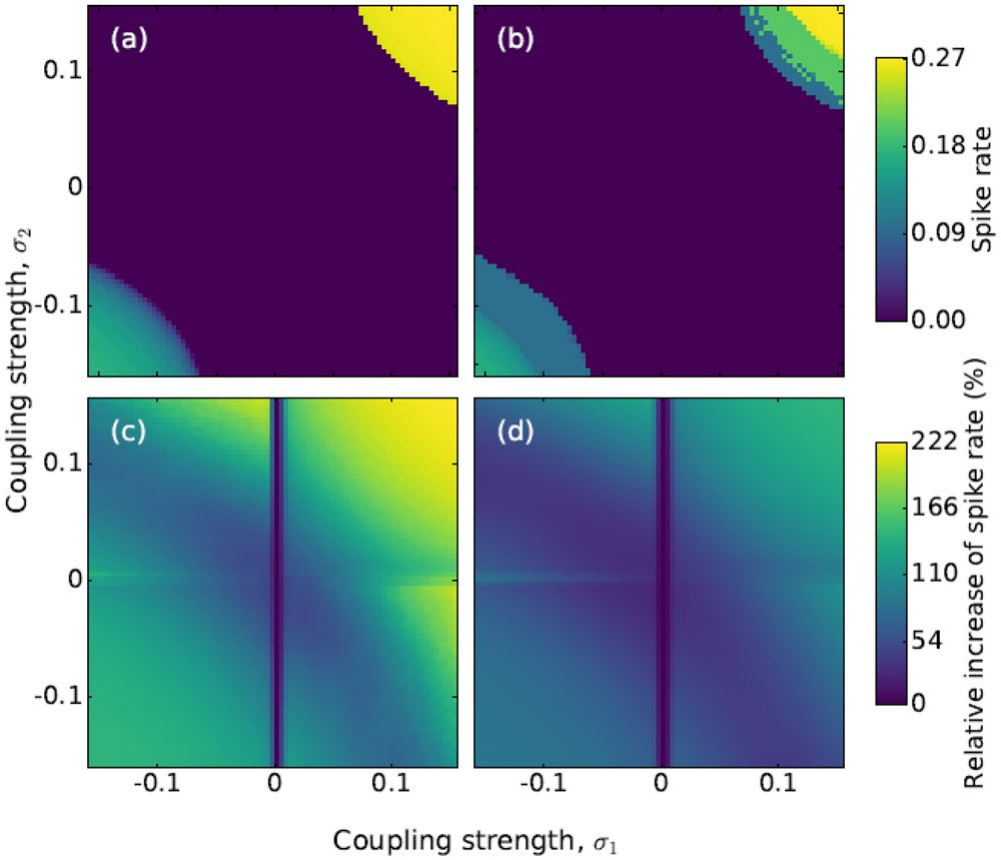
Influence of the coupling strengths in the spike rate. In (**a** and **b**) the deterministic (*D* = 0) spike rate of neuron 1 is plotted in color code, as a function of σ_1_ and σ_2_, when the signal is not applied (*a*_0_ = 0) and when it is applied (*a*_0_ = 0.05 and *T* = 10), respectively. Panels (c and d) display the relative increase of the spike rate (with respect to the uncoupled neuron), when noise is included (*D* = 2 ⋅ 10^−6^). In (**c**) *a*_0_ = 0 while in (**d**), *a*_0_ = 0.05 and *T* = 10.

Having identified the sub-threshold region in the parameter space (*a*_0_, *T*), we next turn our attention to the influence of the coupling coefficients. Figure 3 displays the spike rate as a function of *σ*_1_ and *σ*_2_ in different situations. In panel (a) there is no signal and no noise. We observe that when both |*σ*_1_| and |*σ*_2_| are large enough, the coupling induces spikes. Positive coupling coefficients result in a higher spike rate, in comparison with negative coefficients. In panel (b), the noise is still zero but a weak signal is applied. Because the signal is subthreshold [*a*_0_ = 0.05 and *T* = 10, which are in the sub-threshold region in Fig. 2(a) and (b)], we note only small variations with respect to panel (a).

In Fig. 3(c) and (d) noise is included; in (c) there is no signal while in (d) the weak signal is applied. To show how the spike rate changes with the coupling, Fig. 3(c) and (d) display the relative variation of the spike rate (with respect to the spike rate when neuron 1 is uncoupled). Without signal (panel c), positive coupling coefficients result in larger spike rate as compared to negative ones, however, when the signal is applied (panel d) these differences are washed out. The vertical line in panels (c) and (d) is due to the fact that when *σ*_1_ = 0 neuron 1 is uncoupled from neuron 2, and thus its spike rate does not depend of *σ*_2_.

In order to limit the number of parameters, in the following we assume *σ*_1_ = *σ*_2_ = *σ* and fix (unless otherwise stated) *σ* = 0.05, *a*_0_ = 0.05 and *T* = 10. For these parameters the signal and the coupling act as sub-threshold perturbations: without noise neuron 1 does not fire spikes.

**Figure 4:**
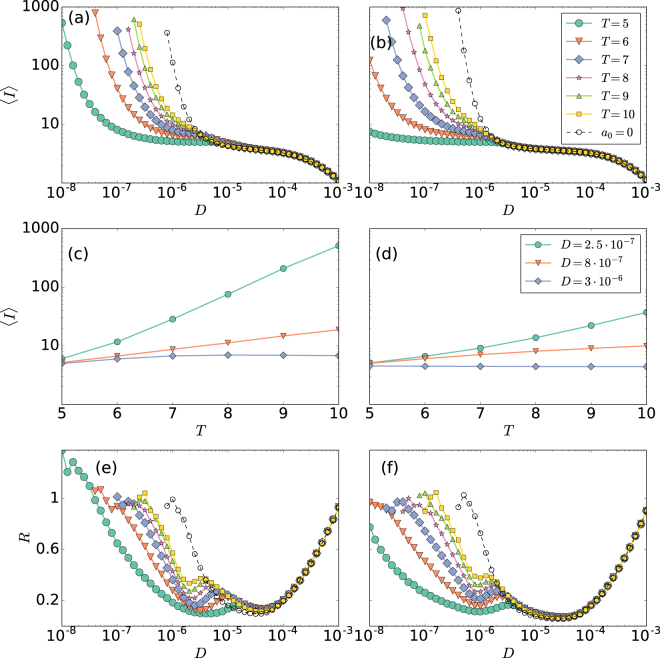
Influence of the noise strength in the mean ISI and in the regularity of the spikes. (**a**,**b**) Mean ISI, 〈*I*〉, of neuron 1 as a function of the noise strength, *D*, for different periods, *T*, of the signal; (**c**,**d**) 〈*I*〉 vs. *T* and (**e**,**f**) ISI normalized standard deviation, *R*, as a function of *D*. Panels (a,c and e) are without coupling (*σ* = 0), while (**b**,**d** and **f**) are with coupling (*σ* = 0.05).

To further characterize the role of noise, Fig. 4 displays the mean ISI, 〈*I*〉, as a function of noise intensity for different periods of the applied signal (in the *Supplementary Information* we analyze the shape of the ISI distribution). In panel (a) *σ* = 0, while in panel (b), *σ* = 0.05. For both cases there is clearly a noise dominated regime, where 〈*I*〉 is the same, regardless of the coupling and of the period of the signal. In contrast, for low noise levels the coupling and the period affect the 〈*I*〉. In panel (a) (*σ* = 0) we can also compare the mean ISI when the signal is applied (solid symbols indicate *a*_0_ ≠ 0 and different periods) and when the signal is not applied (empty circles): we see that, when *a*_0_ ≠ 0 the neuron fires at lower noise intensities as compared to *a*_0_ = 0. Comparing panel (a) with panel (b) (*σ* = 0.05) we note that when neuron 1 is coupled to neuron 2, it starts firing at even lower noise intensities.

Regarding the role of the period of signal, when the noise level is low, the larger *T* is, the larger 〈*I*〉 is. There is a linear relation, as shown in Fig. 4(c) and (d), which holds for both, the coupled and the uncoupled cases. For stronger noise, 〈*I*〉 remains constant when increasing *T*.

Noise-induced regularity in the spike train^30–32^ is characterized in panels (e) and (f), where the normalized standard deviation of the ISI distribution, *R*, is plotted against the noise intensity for different *T*, without and with coupling, respectively. In both panels, two minimums are observed. Whereas the first one indicates stochastic resonance^33–35^, as it occurs when $T \sim \langle I\rangle$, the second one reveals the coherence resonance phenomenon^30,36^, which is independent from the period of the signal. It occurs for an intermediate value of the noise amplitude for which noise-induced oscillations become most coherent. For some periods *T* a maximum appears for very small values of the intensity of the noise. Such maxima are a signature of anticoherence resonance^37^.

After having characterized the effects of the weak signal, of the coupling, and of the noise in the neuron’s spike rate and in the regularity of the spikes, we next turn our attention to the timing of the spikes. We use symbolic ordinal analysis (see *Methods*) to investigate the possible presence, in the ISI sequence, of over expressed and of less expressed spike patterns.

**Figure 5:**
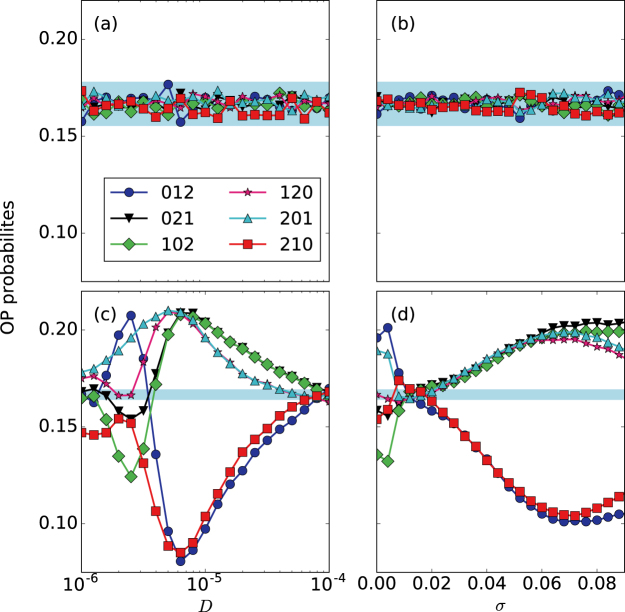
Influence of the noise strength and the coupling strength in the ordinal probabilities. In panels (a and b) the probabilities of the six ordinal patterns are plotted as a function of *D* (for *σ* = 0) and as function of *σ* (for *D* = 2 ⋅ 10^−6^), both for *a*_0_ = 0. Panels (c and d) are as (a and b), but a sub-threshold signal is applied (*a*_0_ = 0.05 and *T* = 10). In all the panels the blue region indicates the interval of probability values that are consistent with the uniform distribution with 99.74% confidence level.

We begin by considering the situation in which no signal is applied and analyze the effect of increasing the noise level or the coupling strength: Fig. 5(a) and (b) display the ordinal probabilities as a function of *D* and *σ*, respectively. We note that neither the noise nor the coupling induce temporal order in the spike sequence (as all the probabilities are within the blue region that indicates values consistent with equal probabilities). When the signal is applied, panels (c) and (d), we note that there is temporal order in the spike sequence, as the ordinal probabilities reveal the presence of over expressed and less expressed spike patterns (the probabilities are not in the blue region and thus, are not consistent with the uniform distribution). Moreover, we note that the variation of the probabilities with *D* or *σ* is qualitatively similar.

**Figure 6:**
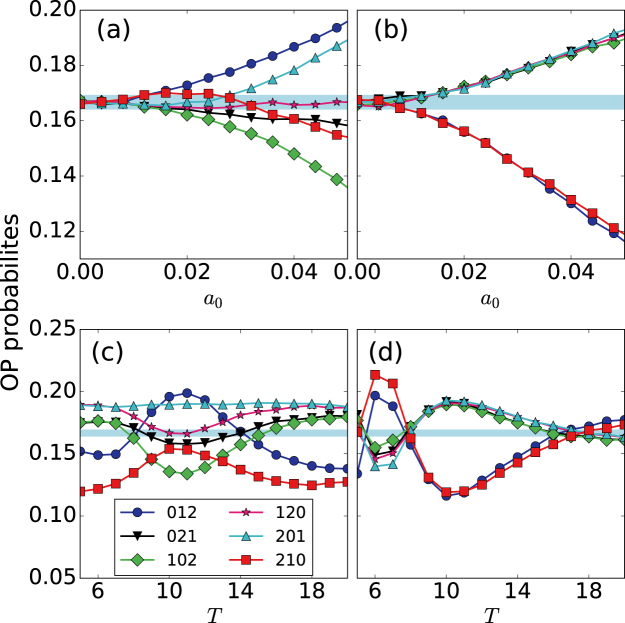
Influence of neuron 2 on the encoding of the signal by neuron 1. (**a**,**b**) Ordinal probabilities vs. the signal amplitude, *a*_0_, when the signal period is *T* = 10; (**c**,**d**) probabilities vs. *T*, when *a*_0_ = 0.05. Panels (a,c) are without coupling (*σ* = 0), while (**b**,**d**) are with coupling (*σ* = 0.05). In all the panels the noise strength is *D* = 2 ⋅ 10^−6^.

Next, we analyze how the coupling coefficients affect the encoding of the signal features (the amplitude and period): we compare how the ordinal probabilities vary with *a*_0_ and *T*, when neuron 1 is isolated [Fig. 6(a) and (c)] and when it is coupled to neuron 2 [Fig. 6(b) and (d)]. In both cases, when *a*_0_ increases (within the sub-threshold region) the probabilities monotonically increase or decrease. This variation is consistent with the results reported in^22^. While in^22^ the sub-threshold signal was applied to the slow variable, *v*, here it is applied to the fast variable, *u*. In both cases, the probabilities encode information of the amplitude of the signal. Nevertheless, coupling to neuron 2 changes the preferred and infrequent patters, i.e., modifies the temporal order in the spike sequence. For instance, for *σ* = 0.05 the probability of the ordinal pattern 012 monotonically increases with *a*_0_, whereas for *σ* = 0.05 monotonically decreases. In panels (b) and (d) we note that, with or without coupling, the preferred and infrequent patterns depend on the period of the signal, confirming the results reported in^22^.

Comparing Fig. 6(c) and (d) we note that the coupling can either improve or degrade the signal encoding with respect to the uncoupled situation: for *T* = 6 and *T* = 10 with coupling (panel d) the probabilities have extreme values (maximum or minimum depending of the ordinal pattern), and thus, the coupling enhances the signal encoding. In contrast, for *T* ~ 17 with coupling (panel d) all the probabilities are close to the blue region (while without coupling they are not), which means that with coupling the probabilities do not encode information of the signal.

Next we investigate if there is an optimal combination of the signal period, *T*, and the coupling coefficients, *σ*_1_ and *σ*_2_, for signal encoding. To quantify the information content of the spike train (represented by symbolic ordinal patterns) we calculate the entropy computed from the probabilities of the ordinal patterns (known as *permutation entropy*, $H=-\,{\sum }_{i}{p}_{i}\,\mathrm{log}\,{p}_{i}$^25^) and normalize the entropy to its maximum value, *H*_*max*_ = −log*L*! with *L*! being the possible number of patterns (see *Methods*).

**Figure 7:**
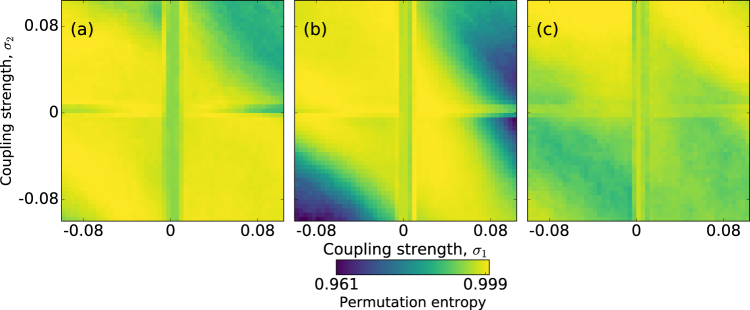
Influence of the coupling strengths on the information content of the spike sequence. The information content is quantified by the normalized permutation entropy that is plotted in color code, as a function of the coupling strengths, *σ*_1_ and *σ*_2_, for three periods of the signal: *T* = 6, 10 and 14, panel (a–c), respectively. Other parameters are: *a*_0_ = 0.05 and *D* = 2 ⋅ 10^−6^.

Figure 7(a)–(c) display the normalized permutation entropy in color code as a function of *σ*_1_ and *σ*_2_ for *T* = 6, *T* = 10 and *T* = 14, respectively. We observe values very close to 1, which indicate that the timing of the spikes is almost random (the ordinal probabilities are almost equal). This is expected as the signal parameters and the coupling strengths are sub-threshold, i.e., the spiking activity is due to the presence of noise (without noise, the neuron displays sub-threshold oscillations). However, for *T* = 10 (panel b) we see that when *σ*_1_*σ*_2_ > 0 the entropy slightly decreases, which indicates that there are more and less expressed patterns in the spike sequence, i.e., the spike sequence carries information about the signal.

**Figure 8:**
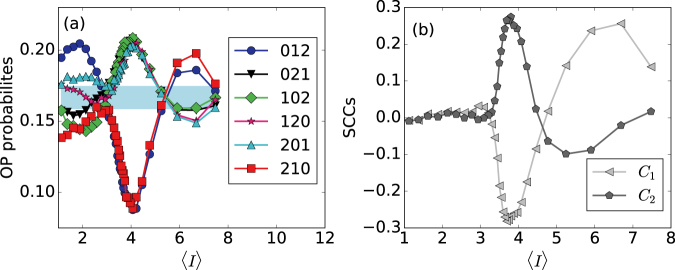
Relation between the ordinal probabilities, the serial correlation coefficients and the mean ISI. (**a**) Ordinal probabilities and (**b**) serial correlation coefficients, *C*_1_ and *C*_2_, as a function of the mean ISI, 〈*I*〉, when the noise strength is varied within the range ${10}^{-6}\le D\le {10}^{-3}$. Other parameters are *a*_0_ = 0.05, *T* = 8, and *σ* = 0.05.

It is interesting to compare the results obtained with nonlinear ordinal analysis, with those obtained with linear analysis. Linear correlations between inter-spike intervals are detected by the serial correlation coefficients (SCCs, see *Methods*). In Fig. 8 the ordinal probabilities and the SCCs are plotted vs. the mean ISI, 〈*I*〉, which is tuned by changing the noise strength [increasing *D* decreases 〈*I*〉 as shown in Fig. 4(a) and (b)]. We see that when the noise is strong (i.e. small 〈*I*〉), the ordinal probabilities are outside the blue region and thus capture temporal ordering in the ISI sequence; in contrast, *C*_1_ and *C*_2_ (that are close to zero) do not capture linear correlations.

**Figure 9:**
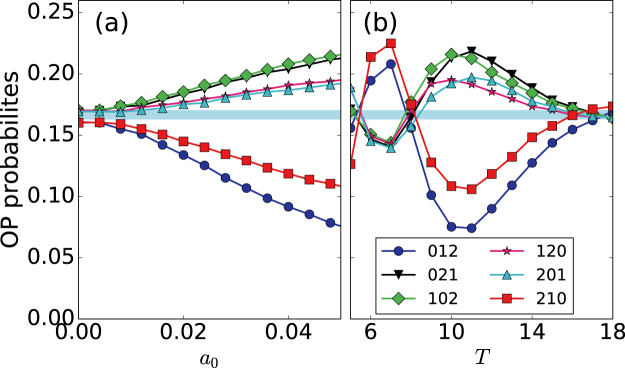
Influence of diffusive coupling on the signal encoding. Panels (a and b) display the ordinal probabilities as a function of *a*_0_ (with *T* = 10) and as a function of *T* (with *a*_0_ = 0.05). Other parameters are *σ* = 0.025 and *D* = 2 ⋅ 10^−6^.

Another relevant issue to discuss is how the coupling terms are implemented. While we have presented model simulations where the terms *σ*_2_*u*_1_ and *σ*_1_*u*_2_ couple neuron 1 to neuron 2 and vice-versa^38^ (see *Methods*), we have also simulated the model with (i) the coupling in the recovery-like variable (i.e., *σ*_2_*v*_1_ and *σ*_1_*v*_2_ added to the rate equations of *v*_2_ and *v*_1_ respectively) and (ii) with differential coupling (i.e., *σ*(*u*_1_ − *u*_2_) and *σ*(*u*_2_ − *u*_1_) added to the rate equations of *u*_1_ and *u*_2_ respectively). We have consistently found that the probabilities of the ordinal patterns vary with both, the period and the amplitude of the signal, in a similar way as with with non diffusive coupling (see Fig. 9).

## Discussion

We have studied two coupled neurons with a weak sub-threshold periodic signal applied to one of them. We have analyzed how the firing activity of the neuron that perceives the signal encodes the signal information, and the role of another neuron that does not perceive the weak signal. We have shown that when the neuron that perceives the signal is coupled to the second neuron, the spike rate increases and the noise level needed for firing spikes decreases, with respect to the uncoupled neuron. We have used symbolic ordinal analysis to investigate temporal ordering in the timing of the spikes fired by the neuron that perceives the signal. We have shown that the spike sequence has over expressed and less expressed ordinal patterns whose probabilities carry information about the features of the signal (the amplitude and the period). We have also shown that, when the noise is strong, the ordinal probabilities can still encode information about the weak signal, which is not encoded in the spike rate (that is independent of the period of the signal) and is not detected by linear correlation analysis (as the serial correlation coefficients at lags 1 and 2 vanish).

Clearly, it is crucial that the neuron that perceives the signal not only encodes the information, but also, transfers the information. In order to investigate information transfer, we plan to analyze, in future work, how the spike sequence of the second neuron (that does not perceive the signal) encodes the information of the signal perceived by the first neuron. In this sense, it is important to compare the dynamical behavior of the second neuron, with and without the applied weak signal, in order to determine which specific property of the spike train (spike rate, SCCs, ordinal probabilities) carry information about the features of the weak signal (amplitude and period) that is perceived by the first neuron. It would also be interesting to analyze the encoding of more complicated signals, for example, a weak signal with two different frequencies, and compare with the phenomenon of vibrational resonance^39^.

Our findings could be relevant for neuronal sensory systems composed by coupled noisy neurons, when only one is affected by external inputs. The encoding mechanism demonstrated here, by which the period and the amplitude of the applied sub-threshold signal are encoded in the values of the ordinal probabilities, is very slow if the probabilities are computed from the spike train of a single neuron, because a large number of spikes are needed in order to compute the patterns’ probabilities. However, if the encoding is performed by neuronal populations, then, the probabilities can be computed from the spikes of many neurons, and in this case, only few spikes per neuron would be enough to compute the probabilities. This ensemble-based encoding mechanism would allow fast encoding of time-varying signals. Ongoing work is devoted to understand the robustness of the proposed signal encoding mechanism when the neuron (or neurons) that perceive the signal is (are) coupled in a small modular network. We are also studying the role of non-Gaussian noise (Poisson), and of synaptic coupling (excitatory or inhibitory). If the encoding mechanism is indeed robust, we then plan to investigate information transmission in large neuronal populations^40–44^.

## Methods

### Model

We consider two identical FitzHugh-Nagumo neurons^23,24^ (in the *Supplementary Information* we present simulations of non-identical neurons), mutually coupled as in^38^, with a periodic signal applied to one of them (referred to as neuron 1):

1 $$\begin{array}{rcl}\varepsilon {\dot{u}}_{1} & = & {u}_{1}-\frac{{u}_{1}^{3}}{3}-{v}_{1}+{a}_{0}\,\cos (2\pi t/T)+{\sigma }_{1}{u}_{2}+\sqrt{2D}{\xi }_{1}(t),\\ {\dot{v}}_{1} & = & {u}_{1}+a,\\ \varepsilon {\dot{u}}_{2} & = & {u}_{2}-\frac{{u}_{2}^{3}}{3}-{v}_{2}+{\sigma }_{2}{u}_{1}+\sqrt{2D}{\xi }_{2}(t)\\ {\dot{v}}_{2} & = & {u}_{2}+a\end{array}$$

The dimensionless variables *u*_*i*_ and *v*_*i*_ are a fast variable that represents the voltage of the membrane, and a recovery-like variable that represents the refractory properties of the membrane (slow variable); *a* and *ε* are parameters that control the spiking activity of the uncoupled neurons. The coupling terms *σ*_2_*u*_1_ and *σ*_1_*u*_2_ mimic synaptic currents from neuron 1 to neuron 2 and vice-versa^38^. The signal has amplitude *a*_0_ and period *T*. The noise is modeled with statistically independent Gaussian white noise terms [〈*ξ*_*i*_(*t*)*ξ*_*i*_(*t*′)〉 = *δ*(*t* − *t*′) and 〈*ξ*_*i*_(*t*)*ξ*_*j*_(*t*)〉 = *δ*(*i* − *j*)] and the noise level, *D*, is the same for both neurons.

The values of the parameters, *a* = 1.05 and *ε* = 0.01, are chosen such that, when *D* = 0 and *σ*_1_ = *σ*_2_ = 0, the neurons are in the excitable regime: each neuron resides in a stable state (rest state) unless it is perturbed. If a strong enough perturbation occurs, the neuron leaves the rest state and after firing a spike, it returns to the rest state. Then, a refractory period follows during which another perturbation will not trigger a spike.

The equations are integrated, starting from random initial conditions, using the Euler-Maruyama method with an integration step of *dt* = 10^−3^. The signal parameters, *a*_0_ and *T*, and the coupling coefficients, *σ*_1_ and *σ*_2_, are varied within the “sub-threshold” region of the parameter space: without noise the voltage-like variables *u*_1_ and *u*_2_ display only small oscillations [see Fig. 1(a)]. For each set of parameters, the voltage-like variable of the neuron that receives the signal, *u*_1_, is analyzed and the ISI sequence is computed, {*I*_*i*_; *I*_*i*_ = *t*_*i*+1_ − *t*_*i*_} with *t*_*i*_ defined by the condition *u*_1_(*t*_*i*_) = 0 considering only the ascensions.

To compute the mean ISI and the coefficient *R* (see below) time series with a minimum number of 100 spikes are generated (as this is sufficient to estimate the mean values of the ISI distribution), while to compute the ordinal probabilities, time series with at least 10000 spikes are generated. This is because a large number of ordinal patterns are needed in order to determine if their probabilities are consistent or not with the uniform distribution^29^.

### Analysis of ISI sequences

The regularity of the ISI sequence is characterized by the coefficient *R*^30^:

2 $$R=\frac{\sqrt{\langle {I}^{2}\rangle -{\langle I\rangle }^{2}}}{\langle I\rangle },$$

where 〈*I*〉 is the mean value of the ISI distribution.

Correlations between ISIs are characterized by the serial correlation coefficients (SCCs):

3 $${C}_{j}=\frac{\langle ({I}_{i}-\langle I\rangle )({I}_{i-j}-\langle I\rangle )}{\langle {I}^{2}\rangle -{\langle I\rangle }^{2}}$$

where *j* is an integer number. SCCs are a standard tool to analyze spike trains, however, they only capture linear correlations. In contrast, a symbolic methodology known as *ordinal analysis*^25^ has been demonstrated to be well suited for detecting nonlinear correlations in spike trains^22,26,29^. In this approach the actual ISI values {*I*_1_, ..., *I*_*i*_, ..., *I*_*N*_} are not taken into account, instead, their relative temporal ordering is considered. Ordinal analysis transforms a particular signal into symbols, which are known as ordinal patterns. Here, ordinal analysis is used to study the spike train of neuron 1: the ISI sequence {*I*_1_, ..., *I*_*i*_, ..., *I*_*N*_} is transformed into a sequence of ordinal patterns, which are defined by the relative order of *L* consecutive ISI values.

Once the length *L* of the ordinal patterns is defined, for each interval *I*_*i*_ the subsequent *L* − 1 intervals are considered and compared. The total number of possible order relations (i.e., ordinal patterns of length *L*) is then equal to the number of permutations *L*!. If we set *L* = 2 we have only two patterns: 12 and 21 for *I*_1_ < *I*_2_ and *I*_1_ > *I*_2_, respectively; if we set *L* = 3, we have 3! = 6 possible ordinal patterns.

The symbolic sequence of ordinal patterns is computed using the function perm indices defined in^45^. Then, the ordinal probabilities are estimated as *p*_*i*_ = *N*_*i*_/*M* where *N*_*i*_ denotes the number of times the i-th pattern occurs in the sequence, and *M* denotes the total number of patterns. If the patterns are equi-probable one can infer that there are no preferred order relations in the timing of the spikes. On the other hand, the presence of frequent (or infrequent) patterns will result into a non-uniform distribution of the ordinal patterns. A binomial test will be used to analyze the significance of preferred and infrequent patterns: if all the ordinal probabilities are within the interval [*p* − 3*σ*_*p*_, *p* + 3*σ*_*p*_] (with *p* = 1/*L*! and ${\sigma }_{p}=\sqrt{p(1-p)/M}$), the probabilities are consistent with the uniform distribution, else, there are significant deviations which reveal the presence of over expressed and less expressed patterns.

Here we use *L* = 3, which allows to investigate order relations among three ISI (i.e., four consecutive spike times). This choice is motivated by the fact that the signal is sub-threshold, i.e., the firing activity of neuron 1 is driven by noise (without noise, there are no spikes). Therefore, only short ISI correlations are expected in the spike train.

## Electronic supplementary material

Supplementary information
