# Supplementary material for: Sub-threshold signal encoding in coupled FitzHugh-Nagumo neurons
Source: Sci Rep. 2018 May 29;8:8276. doi: 10.1038/s41598-018-26618-8 (PMC5974132; doi:10.1038/s41598-018-26618-8)
Supplement: Supplementary file 1 — Supplementary information [file 41598_2018_26618_MOESM1_ESM.pdf]

Supplementary information to "Subthreshold signal encoding in coupled FitzHugh-Nagumo neurons"

Maria Masoliver[1] and Cristina Masoller[1,*]

[1] *Departament de Fisica, Universitat Politecnica de Catalunya, Rambla Sant Nebridi 22, ES-08222 Terrassa, Barcelona, Spain*

Abstract

Here we present results of simulations of two non-identical FitzHugh-Nagumo neurons. We show that the encoding mechanism proposed in the main text (the information of a weak, sub-threshold signal can be encoded in the probabilities of symbolic spike patterns), is robust when the neurons are slightly different. We also discuss how the inter-spike-interval distribution varies with the coupling strength, the amplitude and the period of the signal.

* cristina.masoller@upc.edu

I. ANALYSIS OF COUPLED NON-IDENTICAL NEURONS

In order to check the robustness of our findings when the neurons are not identical, we consider two neurons that have different spike rates, which are controlled by the parameters ϵ_1 and ϵ_2, and different spike thresholds, controlled by the parameters a_1 and a_2. The model equations are:

$$
\begin{aligned}
\epsilon_1 \dot{u_1} &= u_1 - \frac{u_1^3}{3} - v_1 + a_0 \cos(2\pi t/T) + \sigma u_2 + \sqrt{2D}\xi_1(t), \\
\dot{v_1} &= u_1 + a_1, \\
\epsilon_2 \dot{u_2} &= u_2 - \frac{u_2^3}{3} - v_2 + \sigma u_1 + \sqrt{2D}\xi_2(t) \\
\dot{v_2} &= u_2 + a_2.
\end{aligned} \tag{1}
$$

Here a_0 and T are the amplitude and the period of the sinusoidal signal that is applied to neuron 1. σ is the strength of the symmetric mutual coupling (asymmetric coupling coefficients were discussed in the main text).

Figure 1 displays the ordinal probabilities as a function of modulation period, T, for different values of ϵ_1, while keeping constant ϵ_2 (as in the main manuscript, here $\epsilon_2 = 0.01$). We note that the ordinal probabilities depend on the value of ϵ_1 (for example, for large T, patterns 012 and 210 are more expressed for $\epsilon_1 = 0.008$, but they are less expressed for $\epsilon_1 = 0.012$). For higher ϵ_1, as seen in Fig. 1(d), the probabilities encode information only if the period T is short, for large T, the probabilities are all in the gray region (consistent with equally probable patterns). In contrast, as seen in Fig. 2, the ordinal probabilities remain unchanged when ϵ_2 is varied in the range 0.005–0.02, while keeping constant ϵ_1 (as in the main manuscript, here $\epsilon_1 = 0.01$). One can therefore think that there is no effect of the coupling, however, as Fig. 2(c) shows, the ordinal probabilities are very different when neuron 1 is not coupled to neuron 2.

When the values of the parameters a_1 and a_2 are different, the ordinal probabilities also depend on the period of the signal, as shown in Figs. 3 and 4. Taken together, these results indicate that, even if the two neurons are not identical, the period of a weak signal detected by one of the neurons can be encoded in the values of the ordinal probabilities.

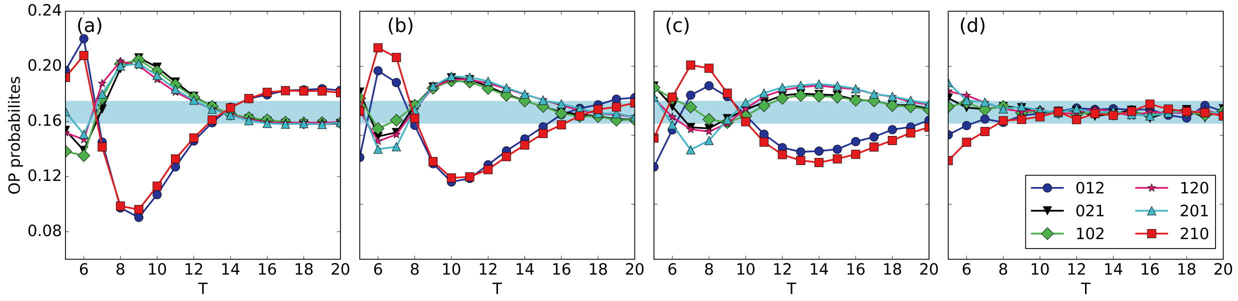

FIG. 1. **Influence of ϵ_1 in the ordinal patterns probabilities.** (a) $\epsilon_1 = 0.008$, (b) $\epsilon_1 = 0.01$ (as in the main text), (c) $\epsilon_1 = 0.012$ and (d) $\epsilon_1 = 0.02$. The other parameters are $\epsilon_2 = 0.01$, $a_1 = a_2 = 1.05$, $a_0 = 0.05$, $D = 2 \cdot 10^{-6}$, and $\sigma = 0.05$.

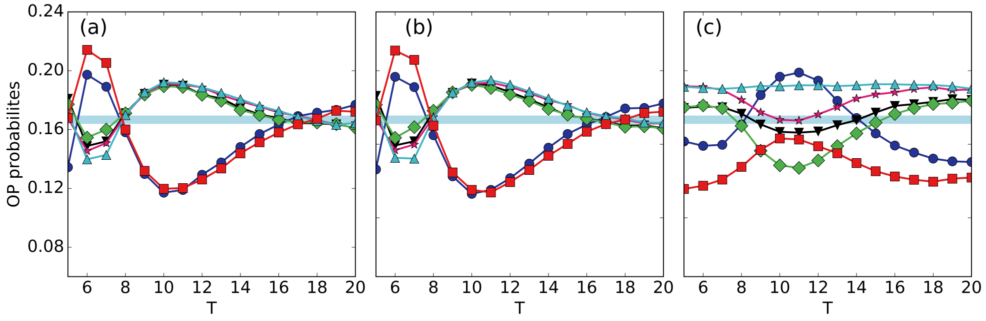

FIG. 2. **Influence of ϵ_2 in the ordinal patterns probabilities.** (a) $\epsilon_2 = 0.005$, (b) $\epsilon_2 = 0.02$. The other parameters are $\epsilon_1 = 0.01$, $a_1 = a_2 = 1.05$, $a_0 = 0.05$, $D = 2 \cdot 10^{-6}$, and $\sigma = 0.05$. In panel (c) the parameters are as in (a) but $\sigma = 0$.

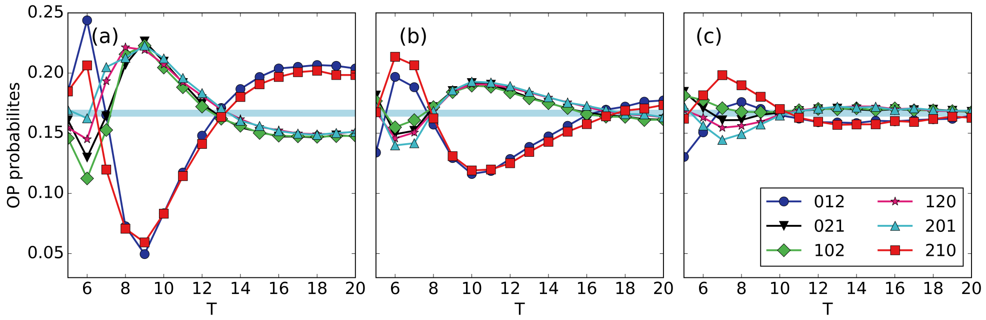

FIG. 3. **Influence of a_1 in the ordinal patterns probabilities.** (a) $a_1 = 1.03$, (b) $a_1 = 1.05$ (as in the main text), (c) $a_1 = 1.07$. The other parameters are $a_2 = 1.05$, $\epsilon_1 = \epsilon_2 = 0.01$, $a_0 = 0.05$, $D = 2 \cdot 10^{-6}$, and $\sigma = 0.05$.

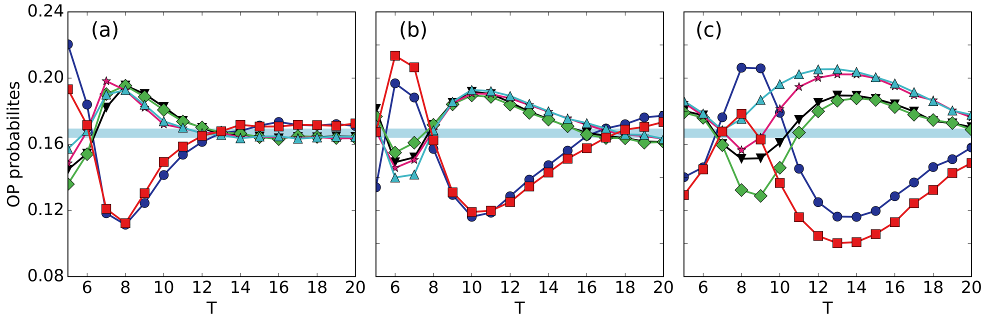

FIG. 4. **Influence of a_2 in the ordinal patterns probabilities.** (a) $a_2 = 1.03$, (b) $a_2 = 1.05$ (as in the main text), (c) $a_2 = 1.07$. The other parameters are $a_1 = 1.05$, $\epsilon_1 = \epsilon_2 = 0.01$, $a_0 = 0.05$, $D = 2 \cdot 10^{-6}$, and $\sigma = 0.05$.

II. ANALYSIS OF THE INTER-SPIKE INTERVAL DISTRIBUTION

In the main text we analyzed the mean inter-spike interval (ISI) and its normalized standard deviation (Fig. 4). Here we discuss how the shape of the ISI distribution depends on the amplitude and on the period of the signal, and how it is affected by the coupling.

Figures 5 and 6 display the ISI distribution for different values of the period and of the amplitude of the signal, respectively. In both figures the left panel corresponds to neuron 1 when is not coupled to neuron 2, and the right panel, when is coupled to neuron 2. The ISI distribution of the uncoupled neuron has a main peak at the period of the signal, T, which becomes more pronounced as the amplitude of the signal, a_0, increases. When the neuron is coupled to neuron 2 (that does not perceive the signal), the peak at T becomes broader and less pronounced. Thus, the coupling to neuron 2 tends to wash out the peak, and thus, if the peak encodes the signal information, the coupling to neuron 2 degrades the signal encoding.

We remark that the results presented in the main text suggest that a different form of signal encoding is plausible, based on how the ordinal probabilities depend on the period and on the amplitude of the signal. We also remark that the information extracted by using ordinal analysis is complementary to that gained by the analysis of the ISI distribution and both, the ordinal probabilities and the ISI distribution can be used for information encoding.

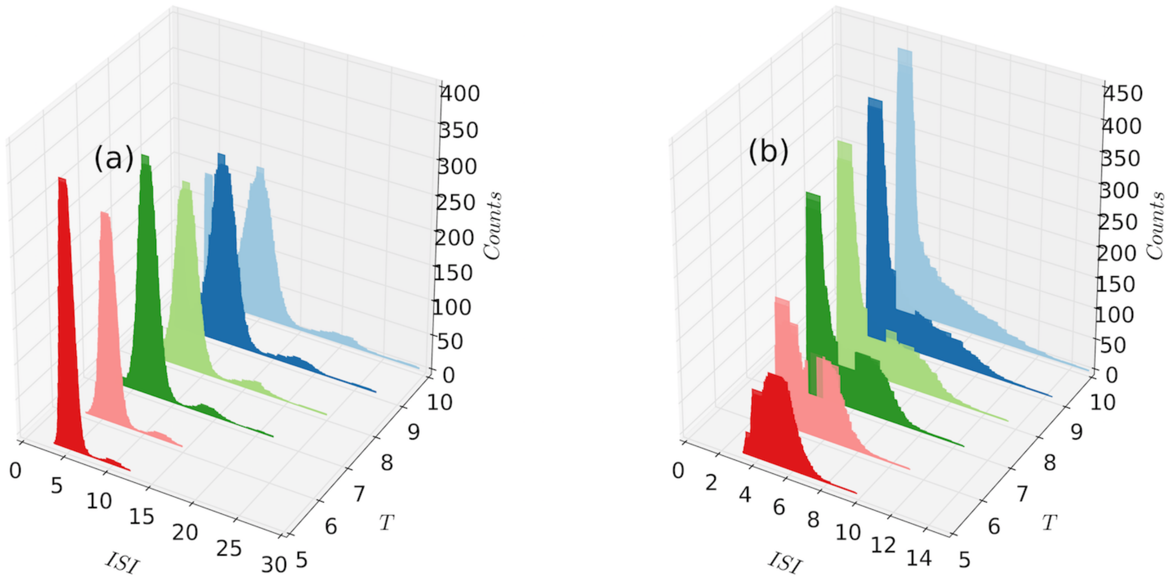

FIG. 5. **Influence of the modulation period on the ISI distribution.** The parameters are $a_0 = 0.05$, $D = 2 \cdot 10^{-6}$ and (a) $\sigma = 0$; (b) $\sigma = 0.05$.

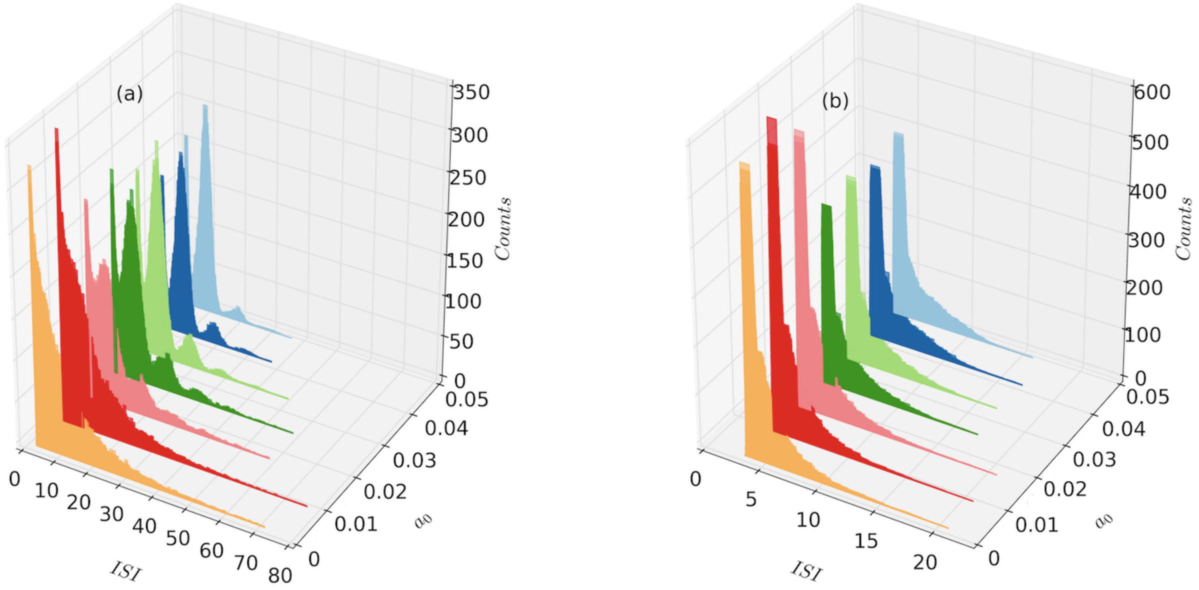

FIG. 6. **Influence of the modulation amplitude on the ISI distribution.** The parameters are $T = 10$, $D = 2 \cdot 10^{-6}$ and (a) $\sigma = 0$; (b) $\sigma = 0.05$.
